# Determination of the through-plane profile of vanadium species in hydrated Nafion studied with micro X-ray absorption near-edge structure spectroscopy – proof of concept

**DOI:** 10.1107/S160057752100905X

**Published:** 2021-11-03

**Authors:** Christian Lutz, Sven Hampel, Sabine Beuermann, Thomas Turek, Ulrich Kunz, Jan Garrevoet, Gerald Falkenberg, Ursula Fittschen

**Affiliations:** a Institute of Inorganic and Analytical Chemistry, Clausthal University of Technology, Arnold-Sommerfeld-Straße 4, Clausthal-Zellerfeld 38678, Germany; b Institute of Technical Chemistry, Clausthal University of Technology, Arnold-Sommerfeld-Straße 4, Clausthal-Zellerfeld 38678, Germany; c Institute of Chemical and Process Engineering Chemistry, Clausthal University of Technology, Leibnizstraße 17, Clausthal-Zellerfeld 38678, Germany; d Energie-Forschungszentrum Niedersachsen, Am Stollen 19A, Goslar 38640, Germany; e Deutsches Elektronen-Synchrotron DESY, Notkestraße 85, Hamburg 22607, Germany

**Keywords:** vanadium redox flow batteries, VRFB, polymer electrolyte membranes, PEM, vanadium speciation, radiation damage, photo-oxidation, water radiolysis, XANES, ionomeric membranes

## Abstract

Vanadium-ion transport through the polymer membrane results in a significant decrease in the capacity of vanadium redox flow batteries. It is assumed that five vanadium species are involved in this process. Micro X-ray absorption near-edge structure spectroscopy (micro-XANES) is a potent method to study chemical reactions during vanadium transport inside the membrane. In this work, protocols for micro-XANES measurements were developed to enable through-plane characterization of the vanadium species in Nafion 117 on beamline P06 of the PETRA III synchrotron radiation facility (DESY, Hamburg, Germany). A Kapton tube diffusion cell with a diameter of 3 mm was constructed. The tube diameter was chosen in order to accommodate laminar flow for cryogenic cooling while allowing easy handling of the cell components by hand. A vertical step size of 2.5 µm and a horizontal step size of 5 µm provided sufficient resolution to resolve the profile and good statistics after summing up horizontal rows of scan points. The beam was confined in the horizontal plane to account for the waviness of the membrane. The diffusion of vanadium ions during measurement was inhibited by the cryogenic cooling. Vanadium oxidation, *e.g.* by water radiolysis (water percentage in the hydrated membrane ∼23 wt%), was mitigated by the cryogenic cooling and by minimizing the dwell time per pixel to 5 ms. Thus, the photo-induced oxidation of V^3+^ in the focused beam could be limited to 10%. In diffusion experiments, Nafion inside the diffusion cell was exposed on one side to V^3+^ electrolyte and on the other side to VO_2_^+^. The ions were allowed to diffuse across the through-plane orientation of the membrane during one of two short defrost times (200 s and 600 s). Subsequent micro-XANES measurements showed the formation of VO^2+^ from V^3+^ and VO_2_^+^ inside the water body of Nafion. This result proves the suitability of the experimental setup as a powerful tool for the determination of the profile of vanadium species in Nafion and other ionomeric membranes.

## Abstract 2

The profiles of vanadium species inside hydrated Nafion membranes have been determined using micro X-ray absorption near-edge structure spectroscopy. This proof of concept study shows how to optimize the procedure with respect to spatial resolution, statistics and species preservation.

## 1. Introduction

Due to their theoretically unlimited capacity and their long life cycle, redox flow batteries (RFB) are promising candidates for short- and long-term energy storage of wind, water and solar power. The most investigated and advanced RFB system is the vanadium redox flow battery (VRFB) (Skyllas-Kazacos *et al.*, 1986 ▸; Weber *et al.*, 2011 ▸; Skyllas-Kazacos *et al.*, 2013 ▸; Noack *et al.*, 2015 ▸; Lourenssen *et al.*, 2019 ▸).

The VRFB consists of two half-cells separated by a polymer electrolyte membrane (PEM). Every half-cell is connected to an electrolyte tank. The negative electrolyte (NE) consists of the redox-active vanadium species V^2+^/V^3+^ and the positive electrolyte (PE) consists of VO_2_^+^/VO^2+^, both dissolved in a 4 *M* sulfuric acid solution. During charging, the electrolytes are pumped continuously through the half-cells, reducing V^3+^ to V^2+^ in the NE and oxidizing VO^2+^ to VO_2_^+^ in the PE. For discharging, the reactions are reversed (Noack *et al.*, 2015 ▸; Lourenssen *et al.*, 2019 ▸).

The performance of the VRFB is mainly determined by the properties of the PEM such as its permeability (Kusoglu & Weber, 2017 ▸). Ideally, it separates the reactive vanadium species and only enables proton conduction. However, the most investigated and used membrane, Nafion, has poor ion selectivity (Schwenzer *et al.*, 2011 ▸). Therefore, vanadium ions are transported through the membrane, often referred to as vanadium crossover. The consequences of this are an im­balance of the vanadium concentration between the two half-cells, self-discharge reactions, and a mainly osmosis-driven water transport. These processes result in a decrease in the capacity over time (Zawodzinski *et al.*, 1993 ▸; Sun *et al.*, 2010 ▸; Schafner *et al.*, 2021 ▸). To make the VRFB more viable and practicable, it is necessary to understand the transport processes through and inside the membrane.

In the last few years, several groups have shown that in a VRFB operated with Nafion as separator, the vanadium concentration increases in the PE and decreases in the NE (Sun *et al.*, 2010 ▸; Luo *et al.*, 2012 ▸). In addition, experimental results and macroscopic observations have been implemented in membrane transport models. VRFB models have been developed by *e.g.* Knehr and co-workers (Knehr *et al.*, 2012 ▸; Knehr & Kumbur, 2012 ▸; Agar *et al.*, 2013 ▸) and Won *et al.* (2015 ▸). Both models are based on the Nernst–Planck equation and consider diffusion, migration and convection. According to their results, the transport is dominated by diffusion. However, the models disagree on the magnitude of the respective transport mechanisms. Redox reactions between the transported vanadium species inside the membrane could be the neglected factor responsible for the deviations of the different models. In our previous work, a proof for redox reactions in the water body of Nafion was found by studying vanadium species in-plane via XANES on the BESSY II BAM*line* (Lutz, Hampel *et al.*, 2021 ▸). The formation of the vanadium dimer V_2_O_3_^3+^ from VO^2+^ and VO_2_^+^ in the PE [*K* = 0.8 *M* ^−1^ (Blanc *et al.*, 1982 ▸)] is another reaction for which we found evidence of occurrence inside the water body of the ionomeric membrane; although the equilibrium constant is quite small, the unique conditions in the water reservoir seem to favor the dimer formation.

Model calculation can predict the profiles of vanadium species once the diffusion coefficients and reaction rates are known. Nonetheless, experimental data for through-plane vanadium transport are necessary to evaluate the validity of the models and to prove that chemical reactions contribute to the transport.

UV–Vis spectroscopy has been used to probe membranes directly (Vijayakumar *et al.*, 2011 ▸). However, the determination of the vanadium species’ composition in the PE with UV–Vis spectroscopy is biased due to the formation of a strongly absorbing dimer (Blanc *et al.*, 1982 ▸). Jia *et al.* (2014 ▸) have shown that synchrotron XANES is suitable for the *in situ* speciation of vanadium in the NE and PE. Compared with UV–Vis, the XANES spectrum of the V *K*-edge (5465 eV) is more specific to the vanadium oxidation state. In the literature, the speciation of vanadium inside PEMs has rarely been addressed and is no doubt more challenging. In our previous work, we showed that laboratory-based and synchrotron XANES are suitable for the speciation of vanadium inside a membrane (Lutz & Fittschen, 2020 ▸; Lutz, Breuckmann *et al.*, 2021 ▸). Laboratory-based XANES is associated with quite a long measurement time (∼5 h per spectrum) and a poor spatial resolution with a probe diameter of the order of millimetres.

The Nafion 117 membrane studied here has a thickness of approximately 180 µm. Hence, the pixel size should not exceed 5 µm to resolve the profiles of the vanadium species. Accordingly, the micro-XANES experiments were performed at a synchrotron radiation source, namely at PETRA III (DESY, Hamburg, Germany). The undulator of the hard X-ray microprobe beamline P06 generates a high-brilliance beam. Even though the X-ray optics, such as the Si(111) crystal monochromator, apertures and filters, and absorption in the 1.5 m long air path between the exit window and the sample eliminate a large fraction of the radiation, the photon flux on the sample is still 6 × 10^8^ photons s^−1^ (the flux without air absorption would be about 2 × 10^10^ photons s^−1^). Since the X-ray beam is focused to an area of 500 nm × 5 µm (V × H, FWHM), the photon flux density is about 2.9 × 10^14^ photons s^−1^ mm^−2^.

It is well known that with increasing photon flux density, the extent of radiation damage increases (George *et al.*, 2012 ▸). Because of this and the high water content of the sample (∼23 wt%), radiation damage by photo-oxidation and water radiolysis is to be expected if no countermeasures are taken. During water radiolysis, reactive species form *e.g.* hydrated electrons ( 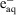 ) and hydroxyl radicals ( 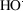 ). Subsequently, they may react with the vanadium ions (Jonah, 1995 ▸). Mesu *et al.* (2007 ▸) studied the influence of X-ray irradiation (photon flux density 1.7 × 10^14^ photons s^−1^ mm^−2^) on organic copper complexes in aqueous solution during XANES analysis. According to their results, the copper changed oxidation state from 2+ to 0 due to water radiolysis. The energy of the Cu *K*-edge shifted to lower energies and the white line decreased. Similar phenomena were observed by George *et al.* (2012 ▸) and Kubin *et al.* (2018 ▸).

Metal ions surrounded by organic ligand molecules, *e.g.* l-histidine in an aqueous solution studied by Mesu *et al.*, and metal ions at the active centers of hydrated proteins described by George *et al.*, have quite similar chemical surroundings to vanadium ions inside the water body of the PEM. Since the vanadium ions studied here are dissolved in the water body system of the PEM surrounded by the sulfonic acid groups of the polymer, species changes due to reaction with hydrated electrons ( 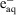 ) and hydroxyl radicals ( 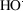 ) comparable with those found in hydrated protein systems are plausible.

It should be noted that radiation damage is not only generated along the track of the primary photon beam through the sample but also by secondary effects like X-ray fluorescence and electron showers due to photoelectrons and Auger electrons (Chapman *et al.*, 2014 ▸). The electron showers are confined to some 10 nm around the primary-beam track for the current low X-ray energy condition (Stuckelberger *et al.*, 2017 ▸). The V *K*-edge X-ray fluorescence (excited for primary-beam energy above the vanadium absorption edge) can transmit through the sample a few 100 µm in all directions, but is comparably weak because of the low vanadium concentration inside the Nafion (∼0.5 wt%) (Lutz, Breuckmann *et al.*, 2021 ▸). Radiation damage to Nafion hydrated with vanadium ions was not observed in laboratory-based XANES (Lutz & Fittschen, 2020 ▸). Further experiments determining species stability with an unfocused beam at BESSY II (photon flux density 2.5 × 10^5^ photons s^−1^ mm^−2^) show that small amounts of V^3+^ are oxidized to VO^2+^ after quite a long irradiation time. According to the results, 5% of the V^3+^ was oxidized after 200 min and at the end of the measurements (700 min) 12% was oxidized (Lutz, Hampel *et al.*, 2021 ▸). The photon fluence for the setup on the BAM*line* was about 1.4 × 10^8^ photons mm^−2^. Since the focused beam on P06 delivers a considerably higher X-ray dose, radiation damage is expected to occur after an exposure time of some milliseconds (photon fluence 3.1 × 10^13^ photons mm^−2^).

In this study, we describe the experimental procedures found to be suitable to study the profile of the vanadium species in Nafion 117. The experimental conditions provide a sufficient vertical spatial resolution and suitable statistics to determine the species but mitigate species alteration (radiation damage) and ion diffusion during measurements. The required spatial resolution and suitable photon flux were achieved by focusing the beam to 500 nm × 5 µm (V × H). The small vertical focus size was chosen to enable spatial resolution along the profile direction. The limitation of the horizontal beam size enables the correction of potential waviness of the sample.

The radiation damage was reduced by keeping the beam horizontally wide (perpendicular to the profile) and the measurement time short (5 ms). The sample was cryogenically cooled to ∼120 K. Due to the low temperature, diffusion processes are inhibited (Ilett *et al.*, 2019 ▸). The minimization of thermal motion is essential as it fixes the profile of the vanadium species. It also freezes the reactive, which are formed from potential water radiolysis. Thereby, the chemical oxidation of the vanadium ions by *e.g.* radicals is inhibited (Le Caër, 2011 ▸; Warkentin & Thorne, 2010 ▸).

In this work, a diffusion cell was constructed which allowed through-plane diffusion from two sides as well as efficient cooling by the Oxford Cryostream. With this cell, profiles of the vanadium species in Nafion 117 membranes could be obtained with a resolution of 2.5 µm. The results show that redox reactions inside Nafion take place during through-plane diffusion. These procedures could perhaps be adapted for the investigation of other ionomeric membranes or X-ray sensitive samples. Furthermore, the obtained experimental data can be used to complete existing VRFB models with respect to transport processes and ion–ion interactions inside the PEM.

## 2. Experimental

### 2.1. Chemicals

Nafion 117 was obtained from Chemours (thickness of the dry membrane 178 µm, equivalent weight 1100 g *n*(SO_3_)^−1^; Wilmington, Delaware, USA). Sulfuric acid (95%–97%) and hydrogen peroxide (30%) were purchased from Merck (for analysis; Darmstadt, Germany). Ultrapure water was generated by a Veolia Elga Purelab Flex 4 water purification system (conductivity 0.055 µS cm^−1^; Paris, France). Vanadium electrolytes were electrochemically converted from V^3+^/VO^2+^ electrolyte (vanadium concentration 1.6 *M*, sulfuric acid concentration 4 *M*; Gesellschaft für Elektrometallurgie mbH, Nürnberg, Germany) using an in-house VRFB cell described by Lutz & Fittschen (2020 ▸). The composition of the vanadium electrolyte was evaluated using a UV–Vis spectrometer (Jasco V-670; Pfungstadt, Germany). The NE was analyzed using a 1 mm quartz cuvette (Hellma, Müllheim, Germany) and the PE using a 0.1 mm flow-through quartz cuvette (Hellma, Müllheim, Germany).

### 2.2. Membrane pretreatment and preparation

Nafion was pretreated similar to the procedure described by Tang *et al.* (2013 ▸). Nafion was held sequentially in 3% hydrogen peroxide, ultrapure water, 1 *M* sulfuric acid and ultrapure water. Every step was performed for 1 h at 353 K.

**Figure 1:**
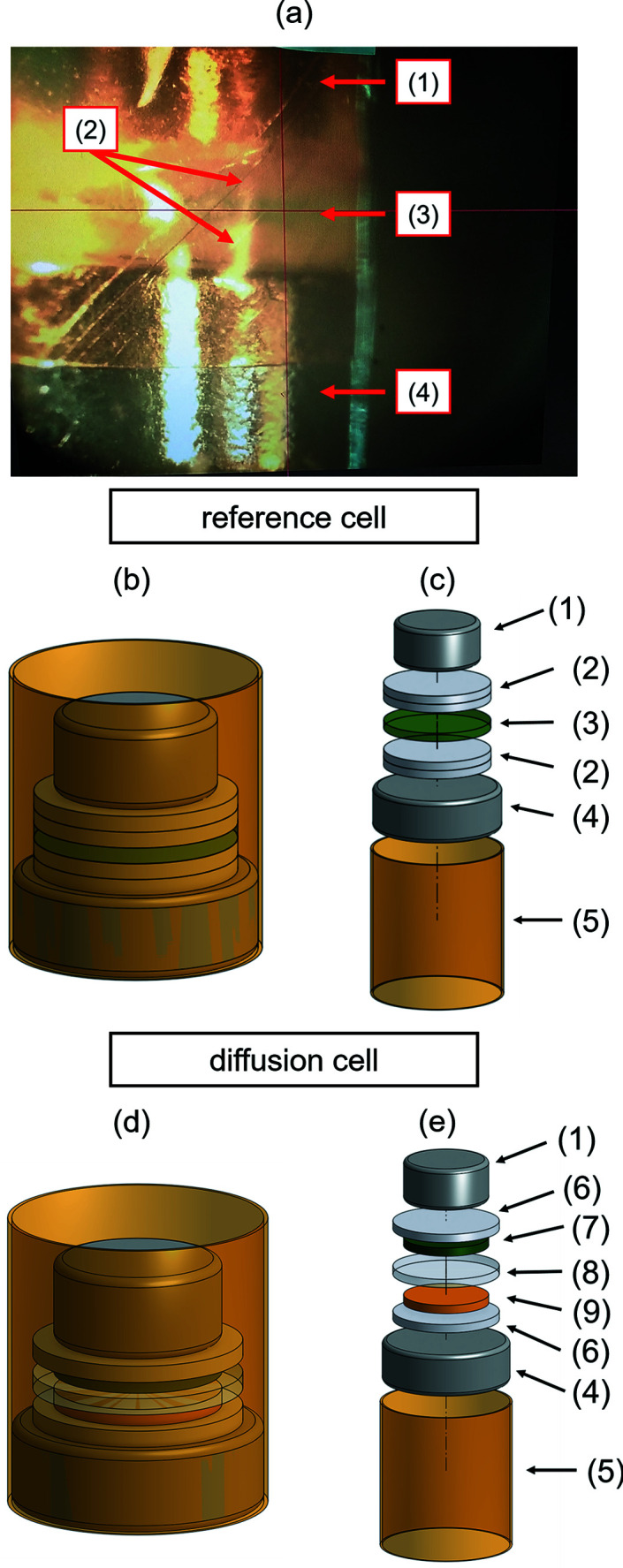
(*a*) A photograph of the sample holder for the measurement of Nafion. (*b*) and (*c*) Render images of the sample holder for the measurement of Nafion (reference), (*b*) normal view and (*c*) exploded view. (*d*) and (*e*) Render images of the sample holder for the measurement of Nafion (diffusion), (*d*) normal view and (*e*) exploded view. In all panels, (1) is the neodymium magnet (diameter 2 mm), (2) two layers of dry filter paper, (3) Nafion 117 soaked with vanadium electrolyte (in the rendering soaked with green V^3+^ electrolyte), (4) neodymium magnet (diameter 3 mm), (5) Kapton tube, (6) one layer of dry filter paper, (7) one layer of filter paper soaked with V^3+^ electrolyte, (8) hydrated Nafion 117 (vanadium-free) and (9) one layer of filter paper soaked with VO_2_^+^ electrolyte.

Pretreated Nafion 117 was immersed in 1.6 *M* electrolyte, either V^3+^, VO^2+^ or VO_2_^+^, for 72 h at room temperature. The membrane was extracted from the electrolyte and superficial electrolyte was removed using a laboratory wipe. A membrane piece with a diameter of 2.5 mm was then stamped out. The membrane piece was placed in a Kapton tube (outer diameter 3 mm, inner diameter 2.94 mm; Goodfellow, London, UK) between two layers of dry filter paper (diameter 2.5 mm, thickness 0.17 mm; Macherey-Nagel, Düren, Germany). The setup was fixed between a neodymium magnet with a diameter of 3 mm at the bottom and a neodymium magnet with a diameter of 2 mm at the top (nickel-coated; magnets4you, Lohr am Main, Germany). A photograph and render images of the described sample holder are shown in Figs. 1 ▸(*a*)–1 ▸(*c*). For the measurements, the samples were mounted on an *xyz* piezo stage.

The diffusion cell was prepared similarly to the membrane sample holder. The differences are that a pretreated and vanadium-free membrane was placed between a filter paper soaked with VO_2_^+^ (diameter 2 mm) at the bottom and a filter paper soaked with V^3+^ (diameter 2 mm) at the top. Both soaked filter papers were surrounded by a larger dry filter paper (diameter 2.5 mm) to prevent leakage of the vanadium electrolyte. In Figs. 1 ▸(*d*) and 1 ▸(*e*), render images of the diffusion cell are presented. For every diffusion experiment, a fresh cell was prepared. The cell was assembled in liquid nitro­gen, removed, and held at room temperature for either 200 or 600 s. During this time, the electrolyte was allowed to defrost and vanadium ions were able to diffuse through the membrane. After the defined time period, the membrane was again frozen in liquid nitro­gen to stop the diffusion and freeze the profile of the vanadium species. Finally, the frozen samples were mounted on the piezo sample stage with cryogenic cooling by an Oxford Cryostream.

### 2.3. Instrumentation

**Figure 2:**
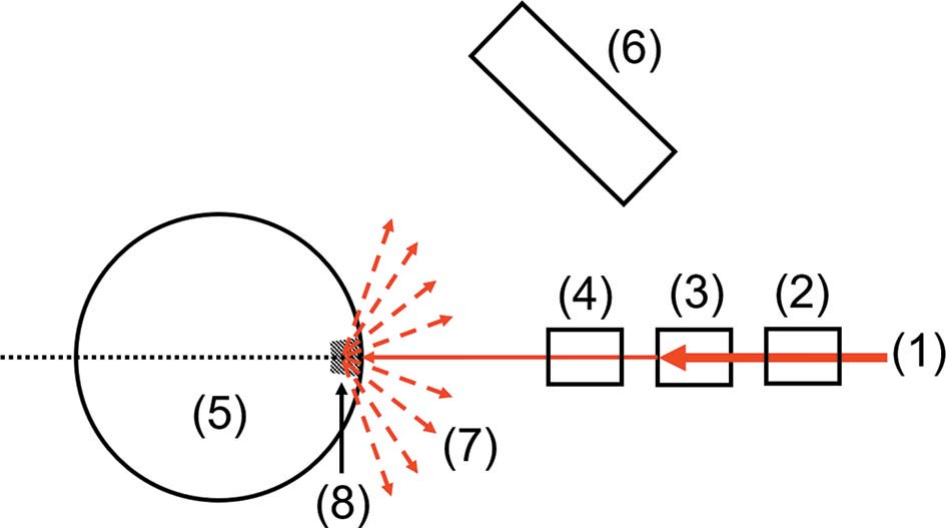
Top-view schematic diagram of the experimental setup at DESY. (1) is the synchrotron X-ray beam, (2) ionization chamber (*I*_0_), (3) Kirk­patrick–Baez mirror system, (4) microscope, (5) sample, (6) Si-drift detector, (7) X-ray fluorescence and (8) excited volume of the sample. Distances are not to scale.

Synchrotron XANES measurements in fluorescence mode were performed on the hard X-ray micro-/nano-probe beamline P06 (PETRA III, DESY, Hamburg, Germany) (Schroer *et al.*, 2010 ▸). The beam was monochromated using an Si(111) double-crystal monochromator (DCM) with an energy resolution of Δ*E*/*E* = 2 × 10^–4^. For higher harmonic suppression, a pair of horizontally deflecting Si mirrors was used. The incoming beam was monitored by a 33 mm long ionization chamber filled with dry nitro­gen. Afterwards, the beam was focused by a Kirkpatrick–Baez mirror system to a size (FWHM) of 0.5 µm × 5 µm (V × H). The characteristic fluorescence radiation was measured with a 50 mm^2^ SII Vortex EM Si-drift detector (Hitachi High-Tech, Chatsworth, California, USA) in 135° geometry, selected to minimize shading effects. According to absorption measurements and calculations with *XOP* (ESRF, Grenoble, France), the attenuation length of the primary beam in hydrated Nafion is of the order of 300 µm and the information depth of the vanadium signal is of the order of 100 µm. The sample was mounted on a three-axis piezo scanner system (Aerotech, Pittsburgh, Pennsylvania, USA) on top of a hexapod (Newport, Irvine, California, USA) for alignment. The piezo scanner system has a working range of 500 µm for every axis. In addition, the sample was cooled to 120 K from above using an Oxford Cryostream Cooler 700 (Oxford, United Kingdom). Because of the cooling, the sample was stabilized against water radiolysis and diffusion of the vanadium ions was stopped. A digital microscope equipped with an HV-Z50W lens (Keyence, Osaka, Japan) was installed between the sample and the Kirkpatrick–Baez mirror system to get a visual overview. A top-view schematic diagram of the setup is shown in Fig. 2 ▸.

**Figure 3:**
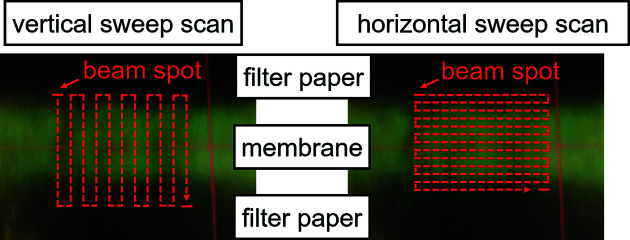
A picture of the membrane in the sample holder with the vertical and horizontal sweep scans indicated.

All measurements were performed in lateral 2D scan mode with continuous movement of the fast axes (sweep scan) and with the energy as the third (slowest) axis. In both sweep scans, the pixel size was 2.5 µm × 5 µm. The vertical fast-axis scan speed was 0.5 mm s^−1^ and the horizontal fast-axis scan speed was 1 mm s^−1^. The acquisition time per 2.5 µm × 5 µm pixel for both scans was 5 ms. However, because of the actual beam dimensions of 500 nm × 5 µm only a sixth of the area was illuminated during the horizontal sweep scans, resulting in a higher local dose compared with the vertical sweep scan, where the entire pixel was illuminated during the same time interval. Hence, the entire area is irradiated homogeneously during vertical sweep scans, while for horizontal scans, between every horizontal line with a height of 500 nm a non-irradiated gap of 2000 nm is present (see Figs. 3 ▸ and 4 ▸). This results in the same total X-ray dose per scan or pixel for both sweep scans, but the local X-ray dose is approximately six times higher for horizontal sweep scans than for vertical sweep scans.

In Figs. 3 ▸ and 4 ▸, the vertical and horizontal sweep scans are illustrated. During vertical sweep scans, an area with beam dimensions of 500 nm × 5 µm is irradiated for 1.68 ms. It experiences an increasing flux for 0.84 ms and a gradually decreasing flux for further 0.84 ms. In total, the area experiences a dose given by the full flux time of 0.84 ms. Analogous to vertical sweep scans, an area of 500 nm × 5 µm is irradiated for 10 ms during horizontal sweep scans and experiences a dose given by the full flux time of 5 ms.

The V *K*-edge spectra were obtained in the energy range 5413–5711 eV. The scan protocol included a sufficient number of data points in the pre- and post-edge region for the normalization. In addition, the pre-edge peak was probed with a small energy step width to allow for optimal species determination. The detailed scan protocol was as follows: 5413–5443 eV, Δ*E* = 10 eV; 5443–5461 eV, Δ*E* = 1 eV; 5461–5479 eV, Δ*E* = 0.33 eV; 5479–5505 eV, Δ*E* = 1 eV; 5505–5571 eV, Δ*E* = 6.4 eV; and 5571–5711 eV, Δ*E* = 20 eV. XANES spectra were obtained from Nafion 117 soaked with a single vanadium electrolyte (V^3+^, VO^2+^ or VO_2_^+^) for reference and from Nafion 117 subjected to diffusion experiments. A vanadium foil (thickness 5 µm; Exafs Materials, Danville, California, USA) served as reference.

The fluorescence spectra were obtained by integrating the fluorescence over 2.5 µm of the vertical sweep scan and over 5 µm of the horizontal sweep scan. The V *K*α signal (*I*) (region of interest) was then added up for every sweep scan pixel and every energy individually and this resulted in a 3D image stack (TIFF files). Simultaneously, the incoming beam intensity (*I*_0_) was measured in the ion chamber and also saved in a 3D stack. From both 3D stacks, either the vertical or horizontal sweep scan pixels of one row were extracted, added up and saved in an ASCII file separately. Then, the added up X-ray spectra (1D scan energy stack) were processed using *ATHENA* (Ravel & Newville, 2005 ▸). A linear combination fit (LCF) in the range 5464–5474 eV (pre-edge peak of V *K*-edge) was performed to assign the vanadium species fractions.

In general, the XANES spectra of one horizontal level (N117 + V^3+^ horizontally, reference spectra of V^3+^ and diffusion experiment) were added up for better statistics. The reference spectra of VO^2+^ and VO_2_^+^, and the spectra of N117 + V^3+^ scanned vertically, were obtained by adding up 60 XANES spectra in the middle of the membrane. The scan settings for all measurements, including the sweep scan direction, the number of added up XANES spectra, the number of vertical steps and the number of horizontal steps, are given in Table 1 ▸.

## 3. Results and discussion

### 3.1. Vanadium speciation in Nafion 117

In this work, a new approach for the determination of the through-plane profile of vanadium species in Nafion using micro-XANES was investigated. However, the speciation of vanadium inside hydrated membranes can easily be hampered by species alteration caused by the brilliant synchrotron microbeam. Hence, the experimental design needed to optimize for (i) minimized alteration of the vanadium species, (ii) spatial resolution sufficient to resolve the profile of the vanadium species, (iii) sufficient statistics and (iv) minimized vanadium diffusion.

The dose was minimized by reducing the flux density and acquisition time. The measurement of a profile requires in principle only a one-dimensionally confined beam. A 2D scan, however, allows us to account for potential waviness of the interface during post-processing of the data. Accordingly, a small vertical dimension for high resolution along the profile (500 nm along 180 µm membrane thickness) and a larger horizontal beam size sufficient to account for waviness (5 µm) were selected. Sweep scanning allowed very short acquisition times per pixel (5 ms). Sufficient statistics were obtained by summing up equivalent spectra collected along the horizontal direction. The horizontal scan length was limited to 500 µm by the scan range of the piezo scanning stage. The short scanning range creates only negligible deviations of the sample–detector geometry.

The experimental setup was designed to accommodate the membrane and the electrolyte reservoirs on both sides of the membrane inside a Kapton tube with a diameter of 3 mm (Fig. 1 ▸). The tiny design was necessary to allow for laminar flow of the Cryostream. The cryogenic temperatures were supposed to minimize diffusion and with that the radiation damage caused by reactive radiolysis products.

The impact of the radiation dose on the quality of the XANES spectra was evaluated by measuring a membrane hydrated with V^3+^ ions with vertical and horizontal sweep scans. The detection of the oxidation of V^3+^ is quite sensitive and the local dose in a horizontal sweep scan is considerably higher. Since the 1*s* → 3*p* transition is not allowed in the octahedral V^3+^, no pre-edge peak in the spectrum of hydrated V^3+^ is present. In contrast, the transition is allowed in the distorted octahedral VO^2+^ and VO_2_^+^. Hence, a pronounced pre-edge peak is only present in the spectra of VO^2+^ and VO_2_^+^ (Wong *et al.*, 1984 ▸; Giuli *et al.*, 2004 ▸; Moretti *et al.*, 2013 ▸; Jia *et al.*, 2014 ▸; Lutz & Fittschen, 2020 ▸; Lutz, Breuckmann *et al.*, 2021 ▸). Previously, we have found that V^3+^ can be oxidized during irradiation to VO^2+^ and the formation of the pre-edge peak is a measure of the stability of the samples towards X-ray radiation. According to the scan speed, beam size and irradiation distribution, the exposure time of every area with the beam dimensions for vertical sweep scans is six times lower than for horizontal sweep scans, as described in the *Experimental* section[Sec sec2]. The X-ray dose *D* was calculated as described by Howells *et al.* (2009 ▸) with the following formula:

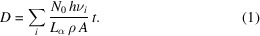

**Figure 5:**
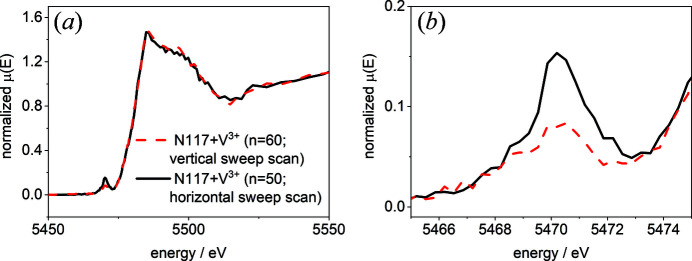
(*a*) The V *K*-edge of Nafion 117 hydrated with 1.6 *M* V^3+^ electrolyte for 72 h measured using vertical (red dashed line) and horizontal (black line) sweep scans. (*b*) An enlargement of the V *K* pre-edge peak region shown in panel (*a*).

Here, *N*_0_ is the photon flux, determined during the measurements (6 × 10^8^  photons s^−1^), *h*ν_*i*_ is the energy of the photons (5413–5711 eV), *L*_α_ is the attenuation length (300 µm), ρ is the density of the sample (1.98 g cm^−3^), *A* is the irradiated area (0.5 µm × 5 µm) and *t* is the exposure time. Accordingly, the local X-ray dose for vertical sweep scans is 3.79 × 10^4^ Gy and that for horizontal sweep scans is 2.26 × 10^5^ Gy. In Fig. 5 ▸, exemplary V *K*-edge spectra for vertical (red dashed line) and horizontal (black line) sweep scans are shown.

The spectra of V^3+^ measured using vertical and horizontal sweep scans differ significantly in the pre-edge peak region. The pre-edge peak intensity in the spectrum measured horizontally is nearly twice that of the pre-edge peak intensity in the spectrum measured vertically. However, the data suggest that the oxidation of V^3+^ occurs already during the vertical sweep scan. Extrapolating from the pre-edge peak intensity, 10% of the V^3+^ are oxidized to VO^2+^ (32.5% in the horizontal sweep scan). The comparison illustrates that, besides the already applied cryogenic cooling and 5 µm broad beam, the exposure time must be kept to a minimum.

Despite the radiation damage, the results prove the reaction of VO_2_^+^ with V^3+^ to VO^2+^ in Nafion and allow the recording of through-plane species profiles, showing *e.g.* a plateau of constant 1:1 VO_2_^+^ to VO^2+^ ratio. The results will be discussed in the following section (Section 3.2). However, for future studies, it is advisable to suppress radiation-induced oxidation even further, so that the profiles of the vanadium species show an even smaller bias. This will be realized by minimizing the X-ray dose. Possibly, a rotation stage instead of an *xyz* stage can be used and the complete circumference of the Kapton tube (9.4 mm) be made available for scanning. This would allow the collection of every data point from a fresh area not irradiated previously, which would lead to an X-ray dose 127 (the number of energy points) times smaller. In view of the information depth of 100 µm (the V *K* X-ray fluorescence attenuation length in Nafion is similar to the attenuation length of the primary beam due to the low vanadium concentration) and no overlap between already irritated volume, the circumference provides ∼1600 data points. Multiple irradiation of volume can be excluded, since the beam penetrates only a fraction of the sample radius. In addition, the scanning speed could be enhanced by increasing the solid angle of detection (*e.g.* Maia detector). Hence, shorter acquisition times are possible, leading to an additional reduction in the local dose.

**Figure 6:**
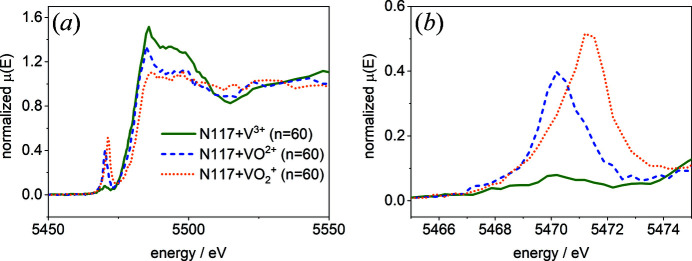
(*a*) The V *K*-edge of Nafion 117 hydrated with 1.6 *M* V^3+^, VO^2+^ and VO_2_^+^ electrolytes for 72 h. (*b*) An enlargement of the V *K* pre-edge peak region shown in panel (*a*).

The reference spectra were obtained from Nafion hydrated with a single vanadium species, taking the precautions described above. In Fig. 6 ▸, the spectra of Nafion hydrated with V^3+^, VO^2+^ and VO_2_^+^ electrolytes are shown.

The spectra of Nafion hydrated with VO^2+^ and VO_2_^+^ electrolytes are comparable with those obtained from *in situ* measurements of vanadium electrolyte by Jia *et al.* (2014 ▸) taken at the Advanced Photon Source (APS), laboratory-based XANES measurements performed by Lutz & Fittschen (2020 ▸), and measurements of Nafion hydrated with vanadium ions by Lutz, Breuckmann *et al.* (2021 ▸) at BESSY II. As expected, the pre-edge region differs significantly for the different vanadium species. The spectrum of VO^2+^ displays a prominent pre-edge peak at 5470 eV and the pre-edge peak of VO_2_^+^ appears at 5471.2 eV. As discussed above, the spectrum of V^3+^ differs in the pre-edge region compared with the literature. However, both the intensity and the energy of the pre-edge peak allow for distinguishing between the vanadium species. In this work, the composition was determined by applying LCF on the pre-edge peak in the energy range 5464–5474 eV. Although the main edge energy increases with higher oxidation state, it was not used for the species determination because (i) more pre- and post-edge energy steps would have been necessary to minimize errors and, maybe most important, (ii) the pre-edge peak does not suffer from self-absorption of the white line, which occurs in fluorescence mode when a sample exceeds the critical thickness. The analyzed samples have an infinite thickness in our experiment and the information depth is in the region of 100 µm.

### 3.2. Determination of in-plane vanadium profile in Nafion 117

**Figure 7:**
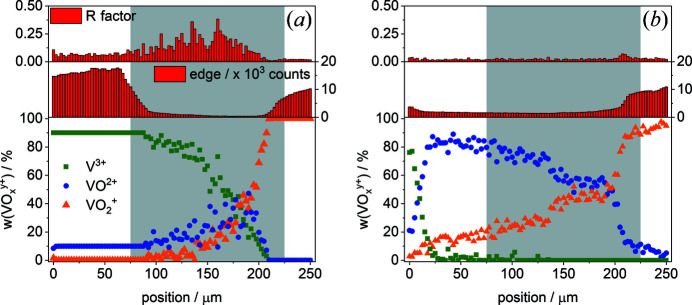
(Top) The *R* factor as a goodness-of-fit parameter. (Middle) The counts of the edge as a function of the membrane position are shown, to represent the total vanadium concentration. (Bottom) The fraction of vanadium species in the membrane: Nafion 117 exposed to V^3+^ from the top and to VO_2_^+^ from the bottom for (*a*) 200 s and (*b*) 600 s. Fractions were obtained from the LCF of the pre-edge peak. Gray areas indicate the position of the membrane.

To study reactions between PE and NE reactive species, the membrane is brought into contact with V^3+^ from the top and with VO_2_^+^ from the bottom, and the ions are allowed to be transported through the plane of the membrane. The advantage of this simple electrolyte combination – in actual VRFBs V^2+^ would also be present in the NE and VO^2+^ in the PE – is that the formation of VO^2+^ unambiguously proves redox reactions occur inside the water body of the membrane. In Fig. 7 ▸, the vanadium profiles are shown for experiments after diffusion times of (*a*) 200 s and (*b*) 600 s. In addition, the counts of the edge (difference in counts before and after the edge) used to estimate the vanadium concentration and the *R* factor as a goodness-of-fit parameter are displayed on the left and right, respectively. The *R* factor was calculated by analogy with Gaur & Shrivastava (2015 ▸) with the following formula:

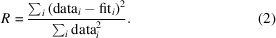




The thickness of the membrane in the diffusion cell is ∼150 µm and is indicated in Fig. 7 ▸ with the gray shaded areas. The adjacent filter papers soaked with vanadium electrolyte (0 µm: V^3+^; 250 µm: VO_2_^+^) were included in the scans (un­colored area in Fig. 7 ▸). After 200 s [Fig. 7 ▸(*a*)], the soaked filter paper shows a high vanadium concentration, which decreases towards the membrane. The membrane has a significantly lower vanadium concentration than the filter papers.

After 200 s [Fig. 7 ▸(*a*)], the V^3+^ fraction in the filter paper soaked with V^3+^ electrolyte and the first ∼10 µm into the membrane is 90% (10% is already oxidized due to radiation damage during the measurements, as discussed above). Further into the membrane, the V^3+^ decreases non-linearly towards the other side of the membrane. A similar profile is observed on the side exposed to VO_2_^+^. However, V^3+^ seems to have diffused faster than VO_2_^+^. This result suggests that the diffusion coefficient of V^3+^ is larger than that of VO_2_^+^. Nevertheless, it should be taken into account that the transport of V^3+^ is additionally supported by gravity. Both vanadium ion fronts meet at approximately 175 µm and form VO^2+^. Here, the highest percentage of newly formed VO^2+^ is found. It decreases towards both ends of the membrane.

**Figure 8:**
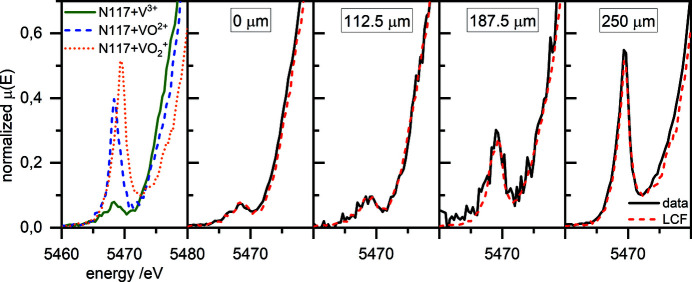
A plot showing the pre-edge peak of the V *K*-edge of Nafion 117 hydrated with 1.6 *M* V^3+^, VO^2+^ and VO_2_^+^ electrolytes for 72 h, together with plots of the pre-edge peak of the V *K*-edge after 200 s at the positions 0, 112.5, 187.5 and 250 µm with the associated fits.

To evaluate the correctness of the data obtained from the LCF, the *R* factor was calculated. The smaller the *R* factor, the better the fit matches the experimental data. After 200 s, the areas with a high vanadium concentration (electrolyte reservoirs) have a small *R* factor of the order of 0.02 (VO_2_^+^) to 0.05 (V^3+^). In contrast, the *R* factor is significantly higher for the fit of data from inside the membrane. With decreasing vanadium concentration, the *R* factor increases. In particular, the interesting regions where the reactions occur have an *R* factor larger than 0.15 and therefore a major error. In Fig. 8 ▸, the fits and experimental data at positions of 0, 112.5, 187.5 and 250 µm are shown. The fit and the experimental data in the electrolyte reservoir (0 and 250 µm) match well. However, the spectra obtained inside the membrane have a low signal-to-noise ratio (Fig. 8 ▸, 112.5 and 187.5 µm).

In general, it is more difficult to distinguish between V^3+^ and VO^2+^ than between VO^2+^ and VO_2_^+^ because V^3+^ and VO^2+^ have the same pre-edge peak energy. In comparison, the determination of the VO^2+^ fraction beside VO_2_^+^ has a smaller error because of the unique pre-edge energy of VO_2_^+^ (Δ = 1.2 eV). The error during VO^2+^ determination is of the order of ∼15% for areas of low concentration (150–200 µm) and decreases with increasing vanadium concentration to less than ∼5%. According to counting statistics, the limit of detection of VO^2+^ beside V^3+^ in the area of low concentration (187.5 µm) is of the order of ∼20%. The detection limit decreases in areas of high concentration (0 µm) down to a value of ∼2%. In conclusion, the concentrations of the formed VO^2+^ are at the detection limit in the 200 s sample. However, the formation of VO^2+^ after 600 s [Fig. 7 ▸(*b*)] supports the presence of VO^2+^ after 200 s.

**Figure 9:**
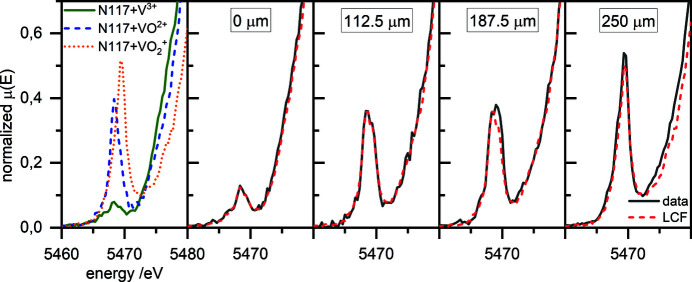
A plot showing the pre-edge peak of the V *K*-edge of Nafion 117 hydrated with 1.6 *M* V^3+^, VO^2+^ and VO_2_^+^ electrolytes for 72 h, together with plots of the pre-edge peak of the V *K*-edge after 600 s at the positions 0, 112.5, 187.5 and 250 µm with the associated fits.

After 600 s [Fig. 7 ▸(*b*)], VO^2+^ has unambiguously formed inside the membrane and diffused to both ends of the membrane. The determination of the vanadium fraction, especially of the VO^2+^ fraction, is more accurate compared with the determination after 200 s because of the quite high vanadium concentration in the whole membrane. Therefore, the *R* factor is quite low, of the order of 0.02, over the entire profile. In Fig. 9 ▸, the fit and experimental data at positions of 0, 112.5, 187.5 and 250 µm are shown. Compared with the spectra of Fig. 8 ▸, the signal-to-noise ratio is higher. According to counting statistics, the limit of detection for VO^2+^ beside V^3+^ is of the order of ∼4% over the complete scanning range. The determination of VO^2+^ beside V^3+^ has an error of ∼5%.

Additionally, the vanadium species and concentration profile after 600 s on the side exposed to VO_2_^+^ shows no significant differences from the one allowed to defrost for 200 s (200–250 µm). However, the fraction of VO^2+^ and VO_2_^+^ has increased into the membrane towards the V^3+^ reservoir. The VO^2+^ percentage increases towards the V^3+^ reservoir from the point where both the V^3+^ and VO_2_^+^ fronts meet. V^3+^ has largely disappeared from the membrane and even from the filter paper. The vanadium ion distribution between 150 and 200 µm is also noteworthy. It seems that over this entire range the ratio of VO^2+^ to VO_2_^+^ is 1:1. It is well known that VO^2+^ and VO_2_^+^ form a dimer at high concentrations (Blanc *et al.*, 1982 ▸). Probably, this interaction takes place inside the membrane and the newly formed dimer acts like a barrier to the diffusion of VO_2_^+^. Hence, the vanadium concentration and vanadium ion profile do not show significant change. The diffusion coefficient of the dimer, to the best of our knowledge, has not been published yet. Our data suggest that it diffuses much more slowly than the other species.

Another phenomenon is observed after 600 s [Fig. 7 ▸(*b*)]: the filter paper exposed to VO_2_^+^ (250 µm) has nearly the same vanadium concentration as after 200 s. However, the other side shows a significant change. The concentrations of the filter paper soaked with V^3+^ and the membrane seem to have equilibrated and the V^3+^ electrolyte is depleted into the membrane. In summary, a set of conditions was determined to study through-plane profiles of vanadium species inside Nafion.

## 4. Conclusions

In this work, it has been shown that synchrotron scanning micro-XANES is a powerful tool for the determination of the vanadium species inside a Nafion membrane. A procedure to study the through-plane profile of the vanadium species in the 180 µm thick Nafion was developed, allowing for a spatial resolution of 2.5 µm. The change in vanadium species was minimized by reducing the mobility of reactive species through cryogenic cooling and by minimizing the radiation dose. It was shown that an increase in the exposure time from 1.68 to 10 ms results in an increase in the vanadium ion oxidation from 10% to 35%.

The profiles of vanadium species were obtained using a diffusion cell setup, where Nafion was exposed from one side to V^3+^ electrolyte and from the other side to VO_2_^+^. The profile of the vanadium species was determined after 200 and 600 s. The orientation of the diffusion was through-plane. For quantification, the pre-edge peak intensity and energy were used. After 200 s the spectra show strong evidence that VO^2+^ was formed. However, the linear combination fits are less reliable than those after 600 s due to the low vanadium concentration inside the membrane. After 600 s the formation of VO^2+^ is evident.

In our previous work, reactions of vanadium ions in the plane of Nafion 117 were observed, albeit with low spatial resolution (Lutz, Breuckmann *et al.*, 2021 ▸). Here, it was shown that this reaction (VO^2+^ is formed from V^3+^ and VO_2_^+^) and the profiles of vanadium species inside the nanoscopic water body of Nafion in the through-plane orientation can be analyzed with a high spatial resolution.

In future work, the diffusion cell will be used to determine the profiles of vanadium species with a higher time resolution and different vanadium electrolyte combinations. The experimental results will then be compared with model results, and *e.g.* diffusion coefficients and reaction kinetics will be determined.

Although the radiation damage was reduced here to a level where meaningful profiles were obtained, the aim of future studies is to reduce the alteration of species further and with that increase the detection efficiency. Therefore, the radiation dose could be minimized by adding a rotation stage and substituting the horizontal scan by a rotational scan for which the Kapton tube needs to be carefully centered. This design is advantageous, because the dose would be spread over a 20 times larger area (circumference of the capillary). When exposed only to a low dose, the same sample can be used for multiple defrosting/diffusion–freezing cycles. In addition, the scanning speed can be increased even more by increasing the solid angle of detection.

## Figures and Tables

**Figure 4:**
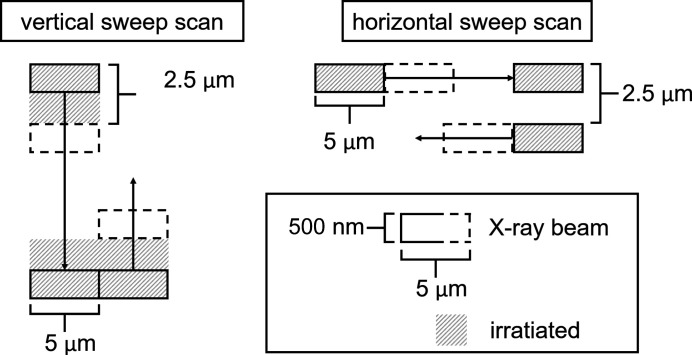
A detailed scheme of the vertical and horizontal sweep scans.

**Table 1:** Summary of the scan settings for all measured samples The columns give the sweep scan direction, the number of added up XANES spectra, the number of vertical steps (beam height 500 nm, step height 2.5 µm) and the number of horizontal steps (beam width 5 µm, step width 5 µm).

Sample	Sweep scan direction	No. of added up XANES spectra	No. of vertical steps	No. of horizontal steps
N117 + V^3+^	Horizontal	50	3	50
	Vertical	60	60	2
N117 + V^3+^ (reference)	Vertical	60	60	60
N117 + VO^2+^ and VO_2_^+^ (reference)		60	60	2
N117 diffusion experiments		60	100	60
